# Comparison of Brain Activation during Motor Imagery and Motor Movement Using fNIRS

**DOI:** 10.1155/2017/5491296

**Published:** 2017-05-04

**Authors:** Alyssa M. Batula, Jesse A. Mark, Youngmoo E. Kim, Hasan Ayaz

**Affiliations:** ^1^Department of Electrical and Computer Engineering, Drexel University, 3141 Chestnut Street, Philadelphia, PA 19104, USA; ^2^School of Biomedical Engineering, Science and Health Systems, Drexel University, 3141 Chestnut Street, Philadelphia, PA 19104, USA; ^3^Department of Family and Community Health, University of Pennsylvania, 3737 Market Street, Philadelphia, PA 19104, USA; ^4^Division of General Pediatrics, Children's Hospital of Philadelphia, 3401 Civic Center Blvd, Philadelphia, PA 19104, USA

## Abstract

Motor-activity-related mental tasks are widely adopted for brain-computer interfaces (BCIs) as they are a natural extension of movement intention, requiring no training to evoke brain activity. The ideal BCI aims to eliminate neuromuscular movement, making motor imagery tasks, or imagined actions with no muscle movement, good candidates. This study explores cortical activation differences between motor imagery and motor execution for both upper and lower limbs using functional near-infrared spectroscopy (fNIRS). Four simple finger- or toe-tapping tasks (left hand, right hand, left foot, and right foot) were performed with both motor imagery and motor execution and compared to resting state. Significant activation was found during all four motor imagery tasks, indicating that they can be detected via fNIRS. Motor execution produced higher activation levels, a faster response, and a different spatial distribution compared to motor imagery, which should be taken into account when designing an imagery-based BCI. When comparing left versus right, upper limb tasks are the most clearly distinguishable, particularly during motor execution. Left and right lower limb activation patterns were found to be highly similar during both imagery and execution, indicating that higher resolution imaging, advanced signal processing, or improved subject training may be required to reliably distinguish them.

## 1. Introduction

Motor imagery is the imagined movement of the body while keeping the muscles still, sometimes considered to be a conscious use of unconscious preparation for an actual movement [1]. There have been numerous studies outlining the similarities between motor execution (overt movement) and motor imagery [2–8]. Of particular interest is whether motor imagery follows the same cortical layout as motor execution in the primary motor cortex (M1). Located in Brodmann's area 4 [9], M1 is subdivided into multiple sections, each responsible for control of a different area of the body, in a layout often referred to as the cortical homunculus [10, 11]. This one-to-one mapping between physical movement and activation in a particular area of the brain provides an opportunity to detect a person's actions (and, potentially, their intended actions) solely through brain recordings, making this an interesting area of brain research. It has also been partially responsible for the popularity of both motor execution and motor imagery as control methods for brain computer interfaces (BCIs), where the mental state is estimated via brain activation patterns [12–26]. Other areas of interest for motor imagery detection are the supplementary motor area and premotor cortex, located anterior to M1 and also involved in the motor network [27, 28].

Many functional magnetic resonance imaging (fMRI) and positron emission tomography (PET) studies have indicated that there is no activation in M1 during motor imagery [29–32] or only limited activation [33–35]. Berman et al. found that training with feedback did not increase motor cortex activity for motor imagery. Additionally, subjects who saw an increase in M1 activation during motor execution feedback training also showed an increase in electromyography (EMG), indicating that the increased fMRI blood-oxygen-level dependent (BOLD) signal may be due to increased muscle activity [29]. In a meta-analysis review, Hétu et al. noted that while motor imagery seems to use similar structures to motor execution, M1 is not consistently activated during motor imagery [35]. Authors nonetheless stress that their findings do not conclusively state that M1 is not involved in motor imagery [35].

It has been proposed that the lack of M1 activation in some motor imagery studies may be due to the lower activation levels produced by motor imagery [36]. An fMRI study by Porro et al. found that motor imagery activated M1 without a significant increase in EMG recordings overall, indicating that the increase in M1 activation was not due to muscle activity [8]. Ehrsson et al. determined using fMRI that hand, foot, and tongue motor imagery follow the same organization as motor execution in M1 [37]. Wriessnegger et al. found significant activation compared to rest for both motor execution and motor imagery in the motor areas, but activation for motor imagery was slower (with an approximately 2-second delay) and smaller in magnitude [38]. While motor execution showed significantly higher activation over the sensorimotor area as compared to the anterior prefrontal areas, motor imagery showed no significant difference in activation between these areas. There were also differences in the activation over time for bilateral and contralateral activation between motor imagery and motor execution. Sitaram et al. also found that fNIRS recordings of motor imagery for left and right hand tapping were similar to motor execution recordings, but smaller in magnitude [23]. An fNIRS pilot study by An et al. compared activation from motor execution, imagery, passive movement, and movement observation for a hand grasping task and found that motor imagery induces a moderate activation in M1 [5].

The type of motor imagery can also affect the quality of motor imagery recordings. Two primary types of motor imagery are visual, where a person self-visualizes the movement, and kinesthetic, where a person imagines the feelings and sensations produced by the movement (i.e., what it “feels like” to perform the motion). Lotze and Halsband suggest that simple, highly kinesthetic tasks may increase M1 activation [27]. An fMRI study by Guillot et al. compared kinesthetic and visual motor imagery in participants with good to excellent motor imagery ability and found that kinesthetic motor imagery shares more similar neural pathways to motor execution, but both forms of motor imagery caused activation in M1 [39].

It has also been reported that motor imagery recordings in M1 are greatly affected by the motor imagery abilities of individual subjects and not just recording and experimental methods [40–42]. The use of objective questionnaires has been proposed in order to determine whether a person will be able to use motor imagery effectively [42]. Miller et al. found that activation in M1 during motor imagery, measured using electrocorticography (ECoG), can be increased via training with feedback and that in some cases it can exceed the original motor execution levels [28]. Similar improvements in motor imagery activation were found when controlling a robot with an electroencephalography (EEG) BCI [43].

A variety of motor imagery techniques have been examined for use with fNIRS. Earlier, Coyle et al. were able to distinguish imagination of squeezing a ball from rest with an accuracy of 70–90% [21]. Other studies have shown up to 89% accuracy distinguishing motor imagery of the left hand or wrist from the right hand or wrist [22, 23]. More recently, fNIRS has been used to detect motor imagery activation for a tennis arm-swinging motion [44] as well as a finger-tapping sequence [45]. The ability to distinguish motor imagery of the feet is also being explored for use in BCIs [12, 24]. While both feet are typically used together in motor imagery BCIs, recently we and other researchers have begun to use left and right feet or legs separately in fNIRS [12, 13] and EEG [46].

In the current study, we aim to explore the similarities and differences between the motor cortex activation recorded via fNIRS during simple motor imagery and motor execution tasks for both upper and lower limb movements. The four tasks under investigation are left hand, right hand, left foot, and right foot tapping as compared to a resting state. To the best of our knowledge, this is the first fNIRS study to compare motor imagery and motor execution for each foot individually. Additionally, we consider the ability of fNIRS to distinguish between left and right foot motor tasks, which could benefit future BCIs through the addition of new control tasks.

## 2. Materials and Methods

### 2.1. Participants

Thirteen healthy participants volunteered in the experiment. Subjects were aged 18–35, right-handed (according to the Edinburgh Handedness Inventory), English speaking, and with vision correctable to 20/20. No subjects reported any physical or neurological disorders or were on medication. The experiment was approved by the Drexel University Institutional Review Board, and subjects were informed of the experimental procedure and provided written consent prior to participating.

### 2.2. Functional Near-Infrared Spectroscopy (fNIRS)

fNIRS is a noninvasive, relatively low-cost, portable, and potentially wireless optical brain-imaging technique [47]. It uses near-infrared light to measure changes in oxygenated (HbO) and deoxygenated (HbR) hemoglobin levels due to the hemodynamic response, the rapid delivery of oxygenated blood to active cortical areas through neurovascular coupling [48]. Recordings from fNIRS are similar to fMRI [49–51], but the measurement area is limited to the outer cortex and has lower spatial resolution (mm versus cm) [49]. However, fNIRS can measure at a higher temporal resolution to capture additional frequency bands and does not require subjects to lay down in a supine position with the loud noises generated by fMRI. Due to their portability, fNIRS devices can be used in more natural settings, such as sitting at a desk and even with mobile participants walking outdoors [52], rather than solely in restrictive and artificial lab environments. Despite the lower temporal resolution and time delay of the hemodynamic response compared to EEG measurements, fNIRS provides a unique trade-off between temporal and spatial resolution and is free from most artifacts, such as muscle activity and eye blinks. It can also easily be used in conjunction with other measurement techniques such as physiological signals [53], EEG [15, 54, 55], and neurostimulation [56, 57].

In the common configuration, light sources and detectors are placed on the scalp and two wavelengths of light are transmitted through the top layer of the cerebral cortex. Light at wavelengths between approximately 700 and 900 nm can pass through skin, bone, and water, but it is absorbed primarily by HbO and HbR [58]. Because HbO and HbR have different light absorption properties, the relative changes in HbO and HbR, and therefore the change in oxygenation of the tissue, can be calculated from changes in the reflected dual-wavelenghth light using the modified Beer-Lambert law [59].

### 2.3. Data Acquisition

Participants sat in a chair facing a monitor that displayed the experiment cues. They were instructed to sit with both feet flat on the floor and hands in their lap or on chair arm rests with palms facing upwards.

**Figure 1:**
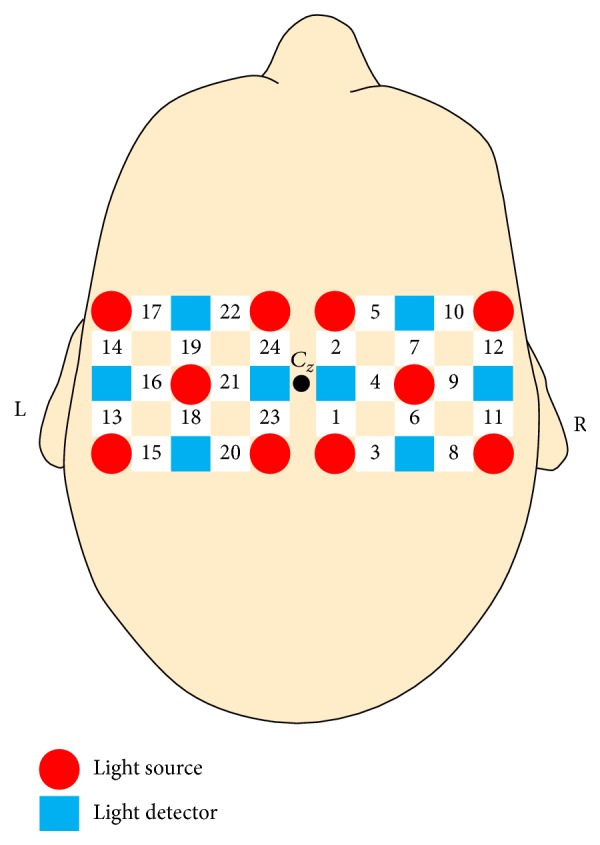
Layout of the light sources, light detectors, and optodes (numbered 1–24). Adjacent sources and detectors are 3 cm apart.

Twenty-four optodes (measurement locations) over the primary and supplementary motor cortices were recorded using a Hitachi ETG-4000 optical topography system, as shown in Figure 1. Sensors were arranged in two separate arrays, one each for the left and right hemispheres. The arrays were placed directly next to each other, and adjacent sources and detectors within each array were 3 cm apart. The center point between the two arrays was aligned with *C*_*z*_ for each participant according to the International 10–20 system. Although not as high spatial resolution as fMRI, fNIRS can provide spatial resolution to capture task differences and distribution of activation over cortical areas comparable to fMRI [49, 51]. HbO and HbR levels were recorded at each location at a sampling rate of 10 Hz.

### 2.4. Experimental Protocol

Motor imagery and motor execution data were recorded on two separate days in order to collect more data while keeping the session length to one hour. Both sessions included motor imagery and motor execution tasks. The protocol included five tasks: rest and tapping of the right hand, left hand, right foot, and left foot. This protocol was developed based on a preliminary study we reported previously [12, 13], and details are provided below.

#### 2.4.1. Tasks

Participants performed five tasks under both motor execution and motor imagery conditions. During motor execution, they were instructed to tap the indicated hand or foot once per second, self-paced. Hand tapping was demonstrated as curling and uncurling the fingers towards the palm, similar to clenching an imaginary ball. Foot tapping kept the heel on the floor as the ankle bent to raise and lower the toes, and subjects were instructed to also curl and uncurl their toes during the movement. During the resting state, participants were instructed to relax, refrain from moving, and not think about anything in particular.

During motor imagery tasks, subjects were instructed to imagine the same actions as performed during motor execution but to refrain from any movement, including muscle twitches. They were also instructed to use kinesthetic imagery, that is, imagine the feelings and sensations felt during an actual movement. Subjects practiced both motor imagery and motor execution tasks, guided by the experimental program, before beginning the experiment in order to familiarize themselves with the protocol and tasks.

**Figure 2:**
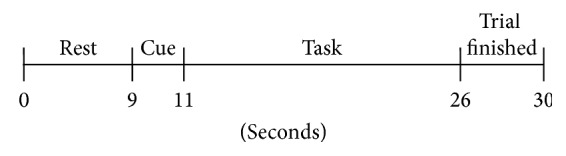
Trial timing diagram.

The trials followed the timing protocol shown in Figure 2. Each trial began with 9 seconds of rest followed by a cue to indicate the upcoming task (e.g., left foot). Subjects then performed the designated task for 15 seconds, followed by a 4-second “Trial Finished” message indicating the task was over. There were a total of 15 seconds between the end of one task and the beginning of another to allow activation to return to a baseline level. Intertrial periods as low as 10 seconds have been used in prior fNIRS motor imagery studies [5, 60, 61].

#### 2.4.2. Session Protocol

**Figure 3:**
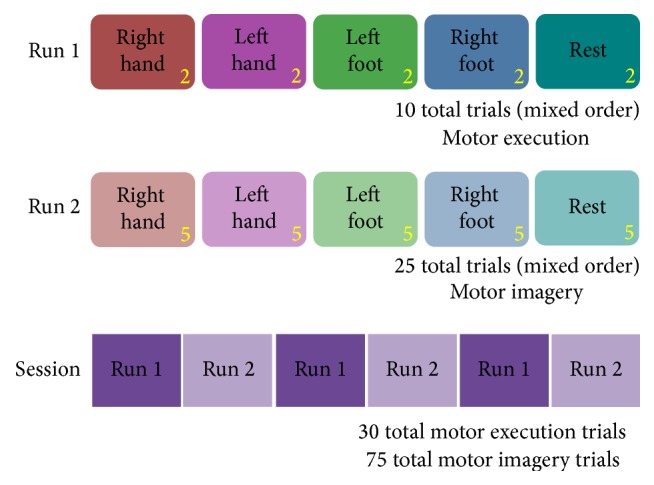
Experiment protocol: each day had three repetitions of the motor execution and motor imagery runs.

Each day was split into multiple runs, as shown in Figure 3. The session consisted of three repetitions of a run with 10 motor execution trials followed by a run with 25 motor imagery trials. The trials in each run (motor imagery or motor execution) contained an equal number of all 5 tasks presented in a pseudorandomized order, without allowing the same task to appear more than twice in a row within each run. Motor execution was interspersed between sections of motor imagery in order to improve the subject's ability to imagine performing the task, both as a reminder of the kinesthetics involved in the actual movement and to reduce fatigue caused by repeated motor imagery trials [62]. A total of 150 motor imagery and 60 motor execution trials were collected for each subject.

### 2.5. Data Analysis

**Figure 4:**
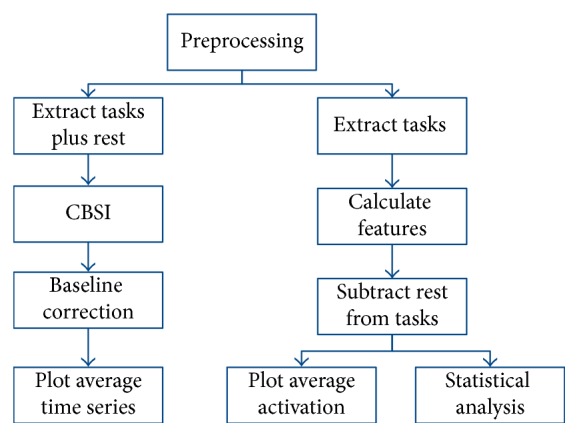
Overview of the data analysis procedure, performed separately for motor imagery and motor execution tasks.

An outline of the analysis procedure, run separately for motor imagery and motor execution data, is shown in Figure 4. All data followed the same preprocessing methods prior to extracting individual task periods for each trial. After preprocessing, there were separate procedures for time series analysis and overall activation and statistical analysis.

#### 2.5.1. Preprocessing

The recorded data were filtered by a 100th order low-pass finite impulse response filter with a 0.1 Hz cutoff frequency in order to remove high-frequency physiological signals such as heart rate and respiration. Data quality was evaluated manually by an expert and optodes with poor quality were removed, as were sections of data that contained artifacts or that were saturated. Subjects needed at least 20 trials of usable data for each of the five tasks, and no more than three optodes missing from both days. Five subjects were excluded due to insufficient data quality. Common average referencing (CAR) based spatial filtering was applied to enhance the signal quality. CAR is a method commonly used in EEG in which the average value of all optodes at each time point is used as a common reference (i.e., the average value across all channels is subtracted from each optode at that time point). This enhances changes in small sets of optodes while removing global spatial trends from the data.

#### 2.5.2. Time Series Visualization

The 15-second task period for each trial was extracted from the data, along with a period of nontask data immediately before and after the trial. Correlation-based signal improvement (CBSI) was applied to each trial period in order to reduce artifact noise and improve signal quality [63]. Baseline correction was then performed on each trial to ensure that the beginning of each task period was approximately zero. This was accomplished by subtracting the average value of the data 0.5 seconds through 0.5 seconds after the start of the task period from every point in the task. The average motor imagery and motor execution time series for a representative optode was calculated for each task.

#### 2.5.3. Average Activation Plots

To create activation plots, the 15-second task period of each trial was extracted immediately after preprocessing. The activation is the difference in HbO levels for each optode between the beginning and end of the trial. To calculate the activation, the average HbO level for the first two seconds of the task was subtracted from the average HbO level from the last 6 seconds of the task (seconds 9–15) for each optode, resulting in a total of 24 features for each trial. The difference in activation level from the beginning of the task was also calculated at multiple other time points during the trial in order to visualize the change in activation over time.

The average activation level for each optode during the resting state was subtracted from all features from that optode, in order to enhance the ways in which the task condition differs from the rest condition. All trials for a given task were averaged across all subjects to give an overall average activation level. These data were arranged into a spatial map according to the optodes' locations and linearly interpolated to show the activation locations.

An additional analysis was performed to show the difference between motor execution and motor imagery by subtracting the average motor imagery values from the motor execution values for the corresponding optode and task.

#### 2.5.4. Statistical Analysis

Statistical analysis was performed using linear mixed models on the average value of the last 6 seconds of each task. Each optode was evaluated for the main effects of task (5 levels: left hand, right hand, left foot, right foot, and rest), type (2 levels: motor imagery, motor execution), and their interaction on the HbO activation level of each optode. Multiple-testing correction (false discovery rate: FDR) was applied to the resulting *p* values for each effect using the R* p.adjust()* function and the “FDR” method. Then, each optode was evaluated for the effect of task on HbO activation levels individually for motor imagery and motor execution (all *p* values adjusted using FDR). Optodes were also evaluated for the effect of type (motor imagery or motor execution) on HbO activation individually for each optode and task (*p* values adjusted using FDR).

An additional post hoc analysis was conducted to determine, for each task, which optodes had a significant increase in HbO levels from the beginning of the task. A linear mixed model compared the average value from seconds 9–15 to the average value from 0-1 seconds individually for each optode and task. The resulting* p* values were also adjusted using FDR.

## 3. Results

Task (5 levels: left hand, right hand, left foot, right foot, and rest) had a significant effect on HbO activation (*p* < 0.05, FDR adjusted) in fifteen optodes (1, 5, 7, 9, 10, 12, 13, 14, 16, 17, 18, 19, 21, 22, and 24). These optodes stretch across the sensory arrays over *C*_*z*_ (according to the International 10/20 system), corresponding roughly to what we expect to find based on the cortical homunculus layout of the motor cortex. Motor type (2 levels: motor imagery, motor execution) showed a significant effect on HbO activation (*p* < 0.05, FDR adjusted) in six optodes (1, 9, 13, 16, 22, and 24), all of which also showed an effect for task. Task and type had a significant interaction (*p* < 0.05, FDR adjusted) for ten optodes (1, 5, 7, 9, 10, 13, 16, 19, 22, and 24), including all six optodes showing an effect for motor type. A table of all results is included in Online Resource 1 (Table S1 in Supplementary Material, available online at https://doi.org/10.1155/2017/5491296).

A post hoc analysis evaluated the effect of task on each optode individually for each motor type. Nine optodes (5, 7, 9, 10, 12, 16, 19, 22, and 24) showed a significant effect of task on motor execution, and three optodes (1, 7, and 22) showed a significant effect of task on motor imagery (*p* < 0.05, FDR corrected). The full table of results is available in Online Resource 1 (Table S2).

**Figure 5:**
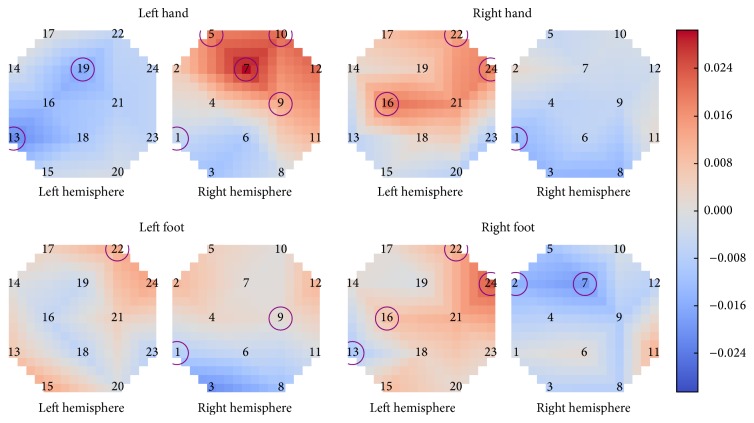
Average difference in activation between motor execution and motor imagery. Optodes with a significant difference (*p* < 0.05, FDR adjusted) between motor execution and motor imagery for a given task are circled.

An additional post hoc analysis examined the difference in HbO activation between motor execution and motor imagery individually for each task and optode. Eleven optodes showed a significant effect of motor type on HbO activation for at least one and up to three of the four different motor tasks (*p* < 0.05, FDR adjusted). Motor execution showed a larger increase in HbO activation than motor imagery on the contralateral hemisphere, particularly for the two hand tasks and right foot task. Additionally, the increased activation for right foot was concentrated more closely in the center, near *C*_*z*_, while the increased activation for hand tasks was further from the center and closer to C3 and C4.  Figure 5 shows the average difference in activation between motor execution and motor imagery for each task, determined by subtracting the average motor imagery activation level from the average motor execution level for each optode. Optodes that showed a significant effect of motor type (*p* < 0.05, FDR adjusted) are circled. The corresponding *p* values are listed in Table S3 of Online Resource 1.

Looking at the average activation levels for motor imagery and motor execution, it can also be seen that motor execution showed a clearer contralateral activation, while motor imagery showed more bilateral activation patterns. The right hand task had the most similar activation pattern between motor imagery and motor execution. Additionally, the right hand motor imagery task has a significant effect on an optode showing a decrease in HbO on the ipsilateral side, but none of the optodes showing an increase in HbO (on either hemisphere) during right hand motor imagery were rated as having a significant effect. Left hand and right foot motor imagery had more bilateral or ipsilateral activation patterns than during motor execution. The right foot task has two optodes that showed significant changes in activation during both motor imagery and motor execution. Additionally, left foot motor imagery shows a more expected activation pattern than for motor execution, with an area of activation and an optode with statistically significant activation in the optodes near *C*_*z*_ on the contralateral side.

Right hand and left hand motor execution tasks are the most easily distinguished among the tasks. Left and right foot motor execution have much more similar activation patterns, although right foot has a more contralateral activation pattern as opposed to that of left foot. Additionally, right hand and right foot motor imagery are very similar, with right foot having slightly lower activation levels and less activation in the optodes further from *C*_*z*_.

**Figure 6:**
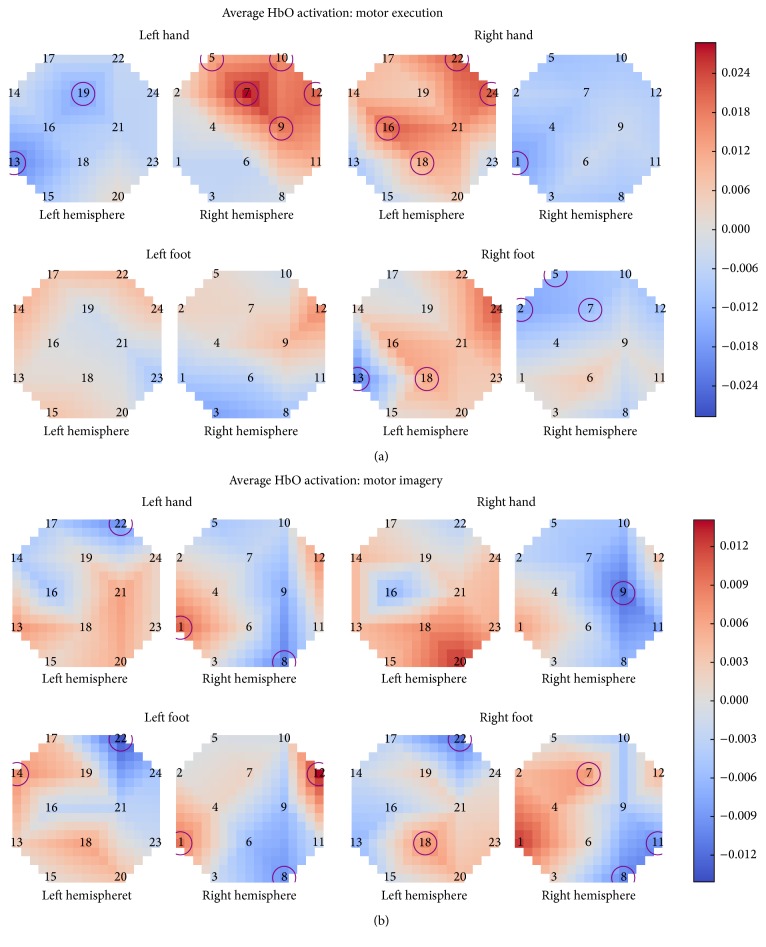
Average HbO activation across all subjects for motor execution (a) and motor imagery (b). Significant optodes (*p* < 0.05, FDR adjusted) are circled.

Figure 6 shows the average HbO activation (contrasted against the resting state by subtracting the rest feature from each of the other tasks for each optode) across all subjects from 9–15 seconds after the start of the task. Optodes found to have a significant (*p* < 0.05, FDR adjusted) difference between the first second of the task and seconds 9–15 are circled.

**Figure 7:**
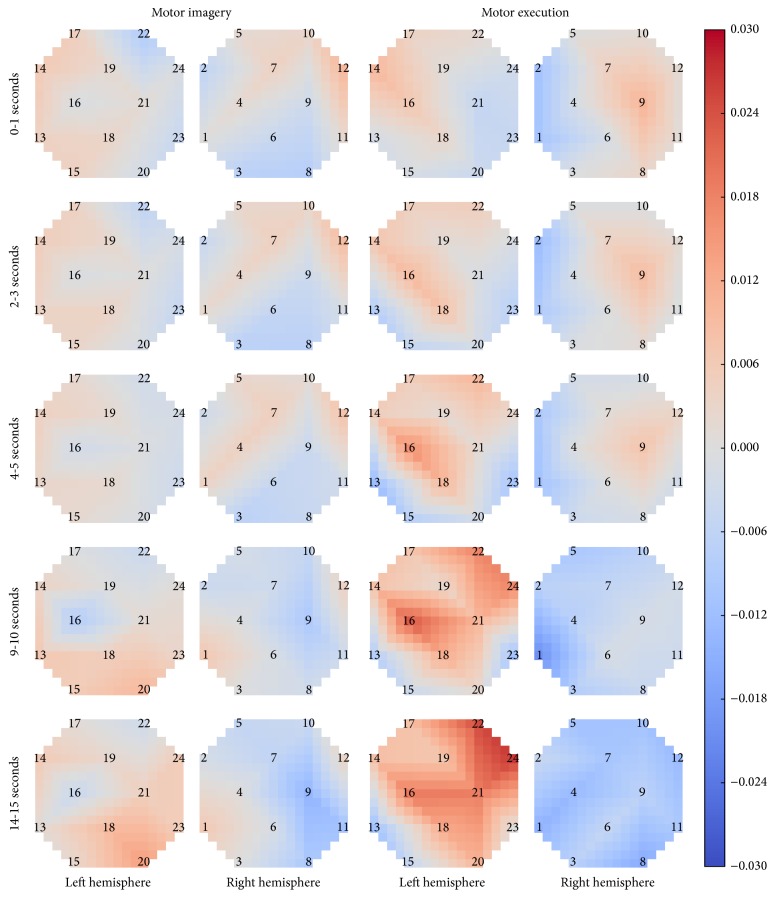
Average in HbO activation over time for motor imagery and motor execution during the right hand task.

The timing of the spatial activation patterns also differs between motor imagery and motor execution. While both motor imagery and motor execution have relatively low, diffuse activation at the start of the right hand task, motor execution quickly shifts to highly contralateral activation (by approximately 5 seconds) and remains mostly unchanged for the duration of the task. Motor imagery shifts to contralateral activation more slowly, by about 10–15 seconds into the task. Additionally, the motor imagery activation levels never reach the strength of motor execution. The timing of HbO activation for the right hand task is shown in Figure 7. Each row shows the average activation over all subjects for a specific time period. Full-size plots for all four motor tasks (left hand, right hand, left foot, and right foot) are available in Online Resource 1 (Figures S1–S4).

**Figure 8:**
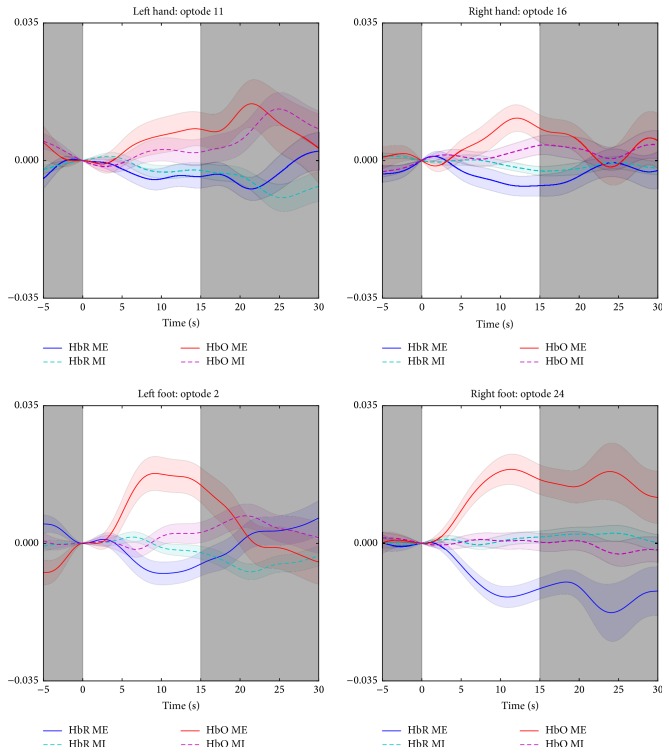
Average HbO and HbR activation for a single optode for each task. Standard error of the mean is shown as a faded area around the average. White area from 0 to 15 seconds is the task period; the grey areas are the resting state before and after the task.

Differences can also be seen in the average time series for a single optode for each of the four tasks. Activation in optode 16 during the right hand task shows a distinct tendency for larger and faster HbO activation during motor execution than imagery, but the latter increases towards the end of the recording period. Left and right foot (in optodes 2 and 24, resp.) show significant activation during motor execution with low or no corresponding activation during motor imagery tasks. In contrast, the left hand shows a more similar activation pattern in both time and strength between motor imagery and motor execution in optode 1.  Figure 8 shows the average time series across all subjects for a single optode during each task, along with the standard error of the mean.

## 4. Discussion

This study examined the differences in brain activity for upper and lower limbs during motor imagery and motor execution tasks recorded using fNIRS. Motor execution and motor imagery showed differences in activation timing of HbO, with motor imagery activation levels increasing more slowly than the corresponding motor execution tasks, as has been reported previously [38]. There were significant differences for spatial distribution of activation between execution and imagery as shown in Figure 5. Moreover, motor execution also showed higher activation levels than motor imagery overall, reflected in the number of optodes with significant HbO activation during the task as well as the number of optodes showing a significant effect of task. These are also in line with previously reported findings [23, 38]. Such differences between execution and imagery could be due in part to the continuous somatosensory and visual feedback of the movement and muscle stimulation that is only present during motor execution [23, 35, 64].

Upper limb (i.e., left and right hand) tasks were the most easily distinguishable for left and right comparison among the four task types based on the spatiotemporal activation patterns. Motor execution hand tasks showed a larger increase in HbO levels, as well as more optodes where task had a significant effect on HbO levels than motor imagery hand tasks. They also showed a strong contralateral activation pattern, while motor imagery hand tasks had more bilateral activation patterns, which has also been observed previously [33].

Right hand demonstrated mostly contralateral activation patterns for both motor imagery and motor execution conditions, as shown in Figure 6, while left hand showed a much more bilateral activation during motor imagery. This could be due to the fact that all participants were right-handed, potentially making the right hand task easier to imagine. Despite its primarily contralateral activation pattern, right hand motor imagery also showed a significant decrease in HbO levels in an optode on the ipsilateral side, without a statistically significant increase for any optodes on the contralateral side. This could indicate that subjects utilized an alternative strategy rather than exactly simulating right hand tapping during the imagery. Future studies may investigate whether training of subjects and use of different mental strategies may affect activity during motor imagery.

Foot tasks had much more bilateral or ipsilateral HbO activation patterns than the hand tasks in both motor execution and motor imagery conditions. Right foot motor execution showed the most contralateral activation pattern, while during motor imagery the activation was much more ipsilateral. However, right foot was the only task to have optodes that showed a statistically significant change from the beginning of the task during both motor imagery and motor execution. The left foot task, on the other hand, showed a more contralateral activation pattern and more optodes with significant activation during motor imagery, while during motor execution the activation was highly diffuse and bilateral, with no significant activation levels at any of the optodes.

The highly bilateral activation patterns during left and right foot tasks indicate that distinguishing between left and right foot using fNIRS may prove difficult. Higher resolution may be required in order to reliably distinguish between the two feet, or they may be best used together in a single “feet” task (as done in some recent BCI studies [17, 65, 66]). Alternatively, using whole leg motor imagery, instead of toe/foot, as in the EEG study by Hsu et al. [46], could cause activation patterns more readily identifiable using fNIRS. Toe and foot motor areas are near or within the longitudinal fissure between brain hemispheres, which is more difficult to measure, while leg motor areas are further apart and closer to the surface of the scalp [10, 11]. In a recent study, we demonstrated the enhanced functional connectivity between motor-related brain regions (M1 and primary somatosensory cortex) and high-level cognitive brain regions during the transition period between rest and hand movements [67]. As dorsolateral prefrontal cortex seems to play a role in the preparation of the sensorimotor system for the task, level of motivation and practice as well as mental workload and distractors in the environment could affect motor imagery related activity levels. One limitation of the current study was the lack of motor imagery ability evaluation of the participants. Future experiments should consider screening subjects for motor imagery abilities as suggested by Marchesotti et al. [42]. Additionally, feedback training could be used to improve motor imagery abilities, as suggested by Miller et al. [28]. This could also improve the distinguishability of the two foot tasks.

## 5. Conclusions

Prior studies have suggested that fNIRS can detect changes in brain activity during motor imagery and motor movement hand tasks similar to fMRI. This study confirms and extends these findings to motor movement and imagery of the left and right foot. Although the activation was relatively weaker, all motor imagery tasks still showed significant levels of activation. The results further suggest that motor execution more strongly evoked the expected contralateral activation patterns compared to motor imagery, particularly in both hand tasks. The differences in spatial distribution of activation between execution and imagery highlights the need for attention when selecting classifier features for BCI use. Moreover, left and right foot activation patterns were more difficult to differentiate than the hand tasks. Differences between left and right foot activation may be made more distinct by using higher resolution imaging, advanced signal processing, such as task-related functional connectivity, or improved subject training with a specific mental strategy.

Current fNIRS-based BCI systems have primarily focused on left/right hand motor imagery tasks. This study opens the door to the use of foot imagery to complement hand imagery tasks in fNIRS-based BCI paradigms. Future BCI systems could develop new approaches to use such multiclass motor imagery to increase overall system performance and BCI usability.

## Supplementary Material

The Supplementary Material accompanying this paper includes additional results in tables for the three statistical analyses. It also includes a figure comparing motor imagery and motor execution activation patterns over time for each of the four motor tasks.
