# Supplementary material for: Comparison of Brain Activation during Motor Imagery and Motor Movement Using fNIRS
Source: Comput Intell Neurosci. 2017 May 4;2017:5491296. doi: 10.1155/2017/5491296 (PMC5435907; doi:10.1155/2017/5491296)
Supplement: Supplementary file 1 — The Supplementary Material accompanying this paper includes additional results in tables for the three statistical analyses. It also includes a figure comparing motor imagery and motor execution activation patterns over time for each of the four motor tasks. [file 5491296.f1.zip › OnlineResource1_CIN_1902840.pdf]

# Comparison of Brain Activation During Motor Imagery and Motor Movement Using fNIRS

## Supplementary Material

Alyssa M. Batula, Jesse A. Mark, Youngmoo E. Kim, Hasan Ayaz

## Contents


- Significance Tests
  - Table S1: Effect of Task, Motor Type, and Task*Type Interaction
  - Table S2: Effect of Task by Optode and Motor Type
  - Table S3: Effect of Motor Type by Task and Optode

- Motor Imagery and Execution Activation Over Time
  - Figure S1: Right Hand
  - Figure S2: Left Hand
  - Figure S3: Right Foot
  - Figure S4: Left Foot

**Table S1** Effect of Task, Motor Type, and Task*Type Interaction. Comparison of Task (5 levels: left hand, right hand, left foot, right foot, rest), Motor Type (2 levels: motor imagery, motor execution), and the interaction between Task and Type. Significant effects ($p$<0.05, FDR adjusted) are highlighted in bold.

| Optode | Task | Type | Task*Type |
|---|---|---|---|
| 1 | **0.0328** | **0.0000** | **0.0329** |
| 2 | 0.2811 | 0.2673 | 0.0637 |
| 3 | 0.2016 | 0.1003 | 0.6258 |
| 4 | 0.4318 | 0.1003 | 0.5784 |
| 5 | **0.0193** | 0.9627 | **0.0072** |
| 6 | 0.0862 | 0.7860 | 0.7322 |
| 7 | **0.0000** | 0.5471 | **0.0000** |
| 8 | 0.2420 | 0.7394 | 0.7997 |
| 9 | **0.0097** | **0.0000** | **0.0105** |
| 10 | **0.0015** | 0.6030 | **0.0113** |
| 11 | 0.1327 | 0.2673 | 0.6462 |
| 12 | **0.0041** | 0.7394 | 0.2157 |
| 13 | **0.0151** | **0.0009** | **0.0399** |
| 14 | **0.0328** | 0.6030 | 0.9796 |
| 15 | 0.4834 | 0.6487 | 0.3599 |
| 16 | **0.0097** | **0.0047** | **0.0019** |
| 17 | **0.0328** | 0.6030 | 0.7997 |
| 18 | **0.0097** | 0.7394 | 0.4848 |
| 19 | **0.0328** | 0.1003 | **0.0329** |
| 20 | 0.7382 | 0.7394 | 0.9796 |
| 21 | **0.0328** | 0.7953 | 0.0566 |
| 22 | **0.0033** | **0.0000** | **0.0065** |
| 23 | 0.2811 | 0.2673 | 0.7202 |
| 24 | **0.0015** | **0.0038** | **0.0065** |

**Table S2** Effect of Task by Optode and Motor Type. Significant effects (p<0.05, FDR corrected) are highlighted in bold.

| | **Motor Execution** | | **Motor Imagery** | |
|---|---|---|---|---|
| **Optode** | **F-Value** | ***p*-Value** | **F-Value** | ***p*-Value** |
| 1 | 2.6811 | 0.0946 | 3.3231 | **0.0448** |
| 2 | 2.2218 | 0.1581 | 0.7146 | 0.7036 |
| 3 | 1.2542 | 0.4309 | 0.1638 | 0.9771 |
| 4 | 1.0478 | 0.5241 | 0.6683 | 0.7036 |
| 5 | 4.5772 | **0.0120** | 0.6441 | 0.7045 |
| 6 | 1.3485 | 0.4295 | 1.2604 | 0.4309 |
| 7 | 12.9852 | **0.0000** | 3.5510 | **0.0332** |
| 8 | 0.5861 | 0.7340 | 1.8726 | 0.2584 |
| 9 | 4.2945 | **0.0143** | 2.6608 | 0.0946 |
| 10 | 6.0101 | **0.0021** | 0.8421 | 0.6297 |
| 11 | 1.2692 | 0.4309 | 1.7141 | 0.3153 |
| 12 | 4.1872 | **0.0150** | 1.3980 | 0.4295 |
| 13 | 2.9167 | 0.0732 | 2.2381 | 0.1581 |
| 14 | 0.9764 | 0.5608 | 2.5690 | 0.1040 |
| 15 | 1.0897 | 0.5098 | 0.4512 | 0.8230 |
| 16 | 5.8867 | 0.0021 | 0.6750 | 0.7036 |
| 17 | 1.5853 | 0.3659 | 1.3229 | 0.4295 |
| 18 | 2.4592 | 0.1201 | 1.5325 | 0.3659 |
| 19 | 3.2432 | **0.0495** | 0.2530 | 0.9473 |
| 20 | 0.0588 | 0.9936 | 0.9298 | 0.5783 |
| 21 | 3.1099 | 0.0566 | 1.5488 | 0.3659 |
| 22 | 4.7873 | **0.0106** | 4.0439 | **0.0157** |
| 23 | 1.1272 | 0.4994 | 0.6661 | 0.7036 |
| 24 | 4.4088 | **0.0133** | 1.3407 | 0.4295 |

**Table S3** Effect of Motor Type by Task and Optode. Optodes and Tasks with a significant effect of Motor Type ($p<0.05$, FDR adjusted) are shown in bold. The tasks are Left Hand (LH), Left Foot (LF), Right Foot (RF), and Right Hand (RH).

| **Optodes** | **LH** | **LF** | **RF** | **RH** |
|---|---|---|---|---|
| 1 | **0.0099** | **0.0019** | 0.0709 | **0.0001** |
| 2 | 0.9573 | 0.7512 | **0.0011** | 0.7798 |
| 3 | 0.6287 | 0.1023 | 0.9311 | 0.4473 |
| 4 | 0.8615 | 0.9252 | 0.1023 | 0.1161 |
| 5 | **0.0064** | 0.7798 | 0.0675 | 0.4098 |
| 6 | 0.4473 | 0.9228 | 0.5394 | 0.8151 |
| 7 | **0.0000** | 0.9073 | **0.0010** | 0.8151 |
| 8 | 0.4098 | 0.9252 | 0.8611 | 0.7798 |
| 9 | **0.0001** | **0.0093** | 0.5702 | 0.2952 |
| 10 | **0.0008** | 0.8615 | 0.6503 | 0.6780 |
| 11 | 0.3452 | 0.9073 | 0.1947 | 0.9073 |
| 12 | 0.2466 | 0.9073 | 0.1070 | 0.5266 |
| 13 | **0.0005** | 0.9823 | 0.1023 | 0.1161 |
| 14 | 0.9252 | 0.8521 | 0.6882 | 0.7755 |
| 15 | 0.2250 | 0.5394 | 0.8151 | 0.4473 |
| 16 | 0.6705 | 0.8611 | **0.0399** | **0.0000** |
| 17 | 0.9073 | 0.7798 | 0.9823 | 0.3205 |
| 18 | 0.2810 | 0.5394 | 0.6503 | 0.6882 |
| 19 | **0.0020** | 0.4473 | 0.7798 | 0.6503 |
| 20 | 0.9073 | 0.9073 | 0.9573 | 0.7022 |
| 21 | 0.0709 | 0.9823 | 0.4473 | 0.2120 |
| 22 | 0.6705 | **0.0013** | **0.0005** | **0.0002** |
| 23 | 0.5369 | 0.5394 | 0.8615 | 0.1947 |
| 24 | 0.3157 | 0.1330 | **0.0064** | **0.0095** |

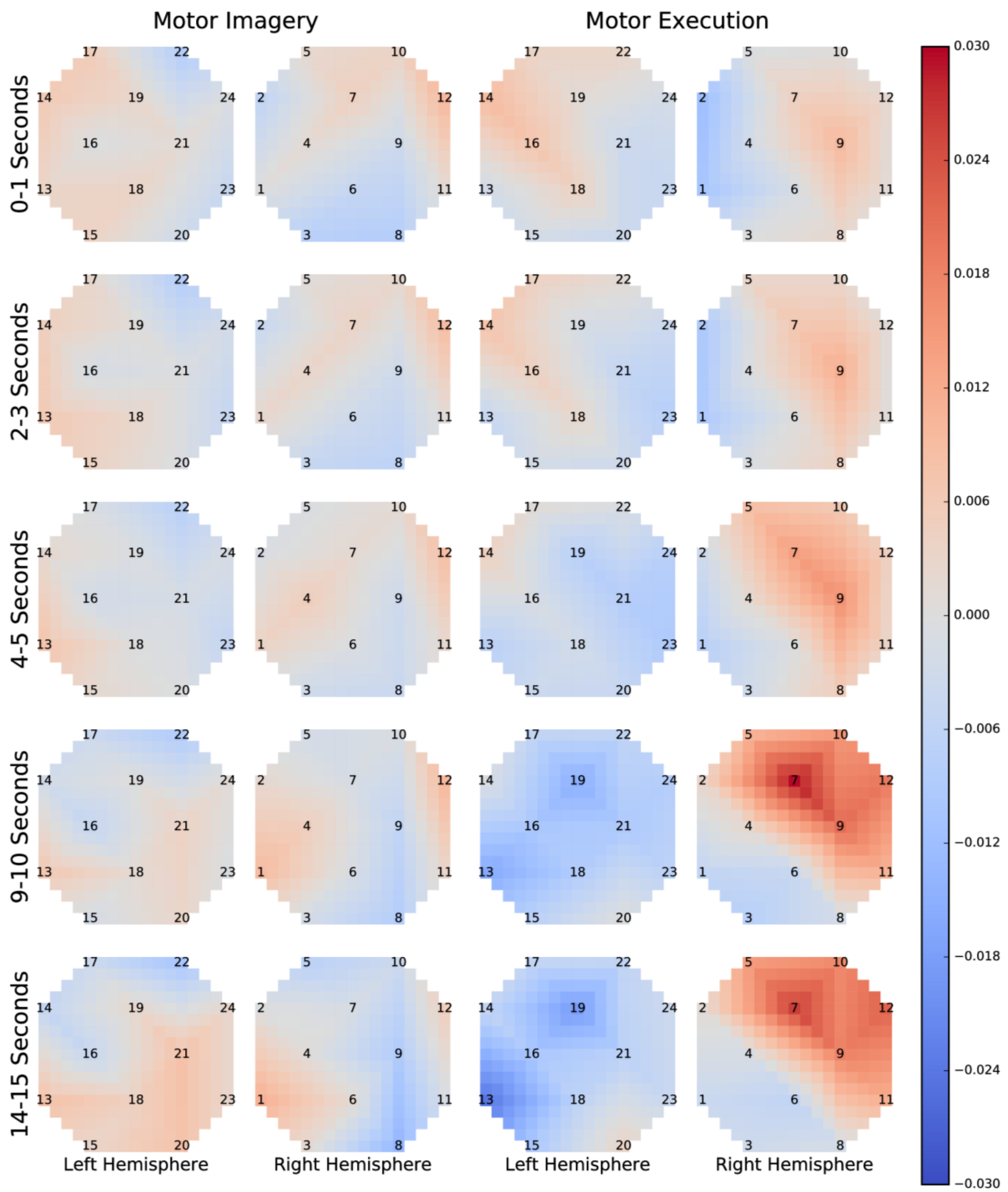


**Fig. S1** Average in HbO activation over time for motor imagery and motor execution during the left hand task

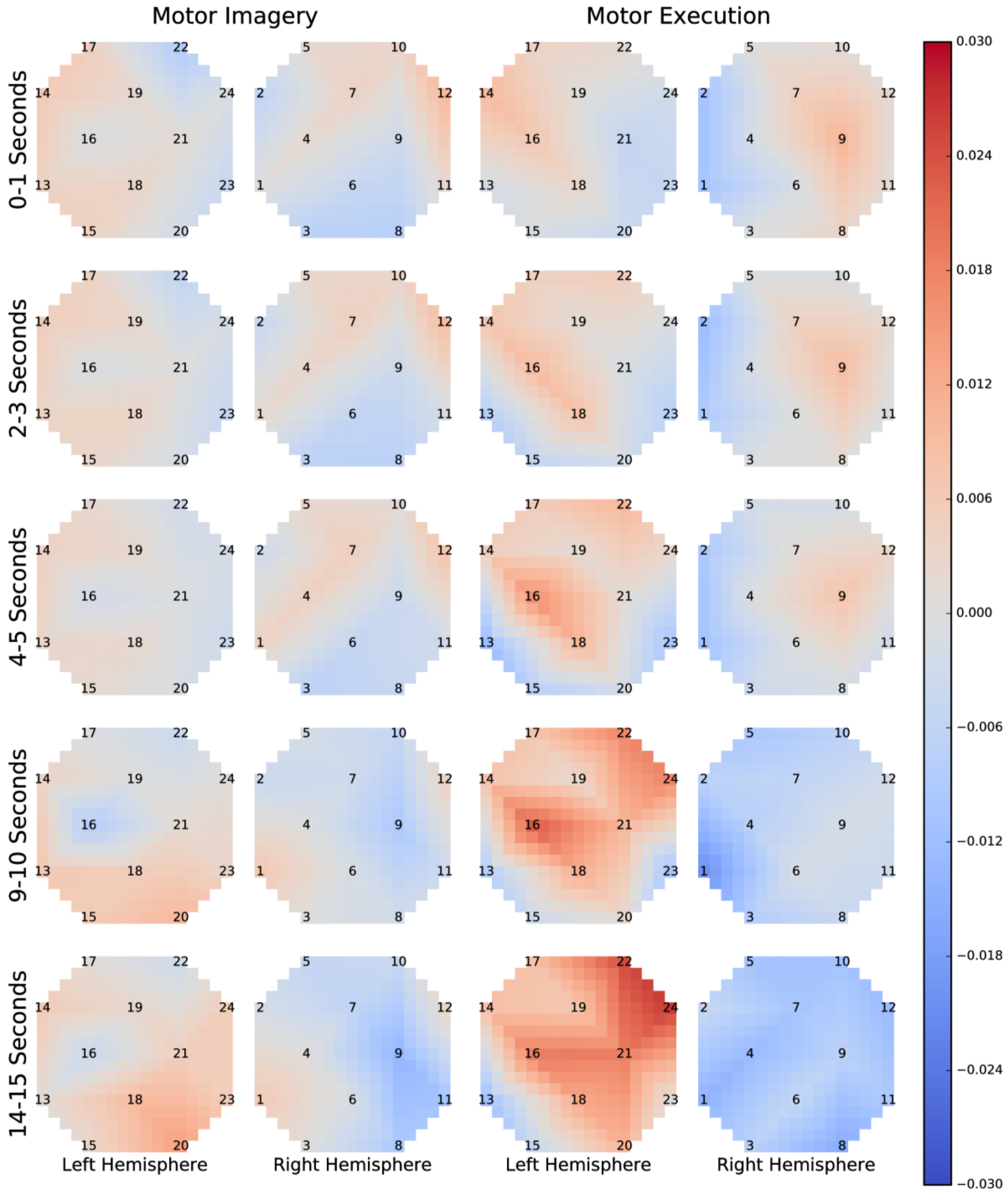


**Fig. S2** Average in HbO activation over time for motor imagery and motor execution during the right hand task

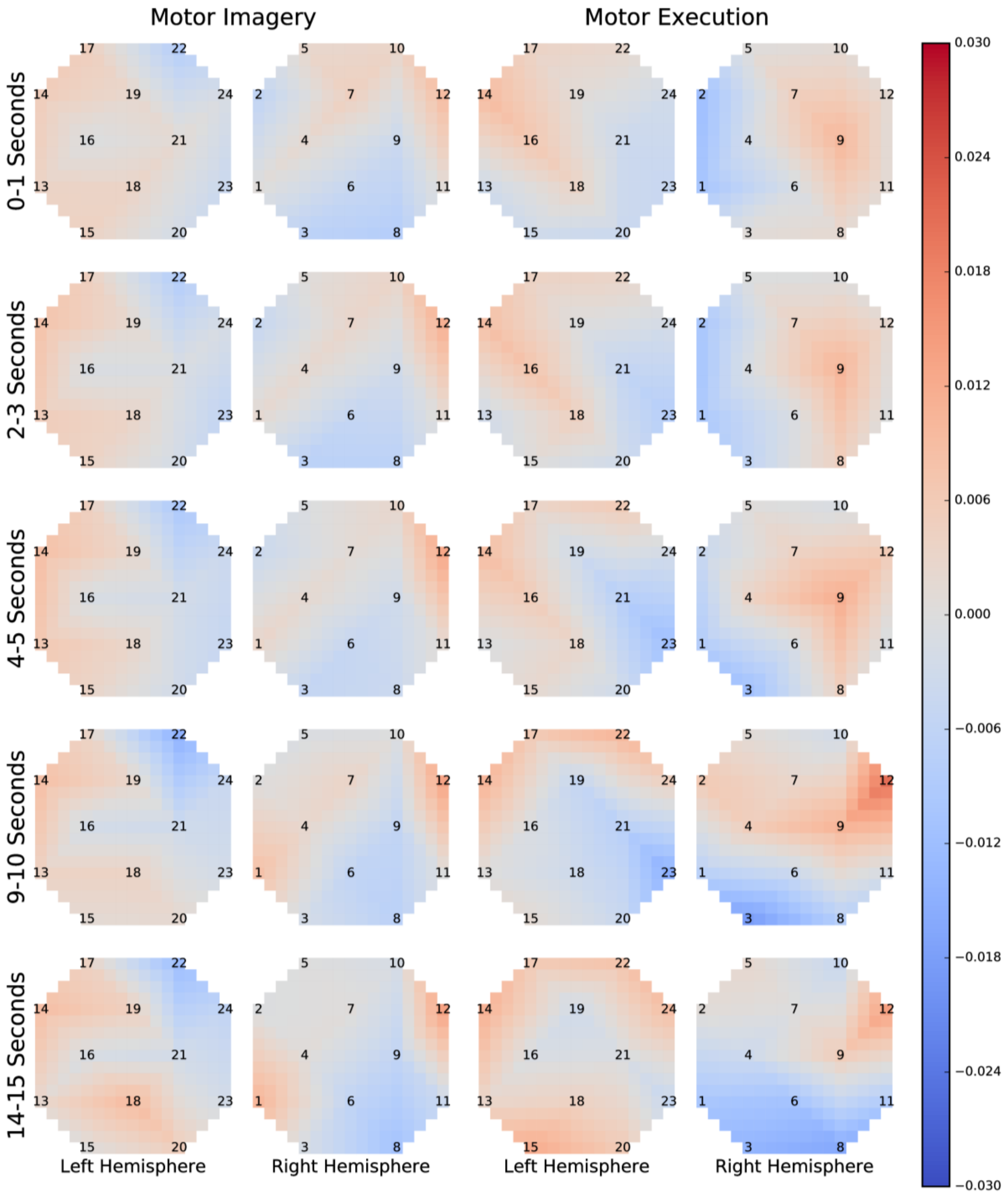


**Fig. S3** Average in HbO activation over time for motor imagery and motor execution during the left foot task

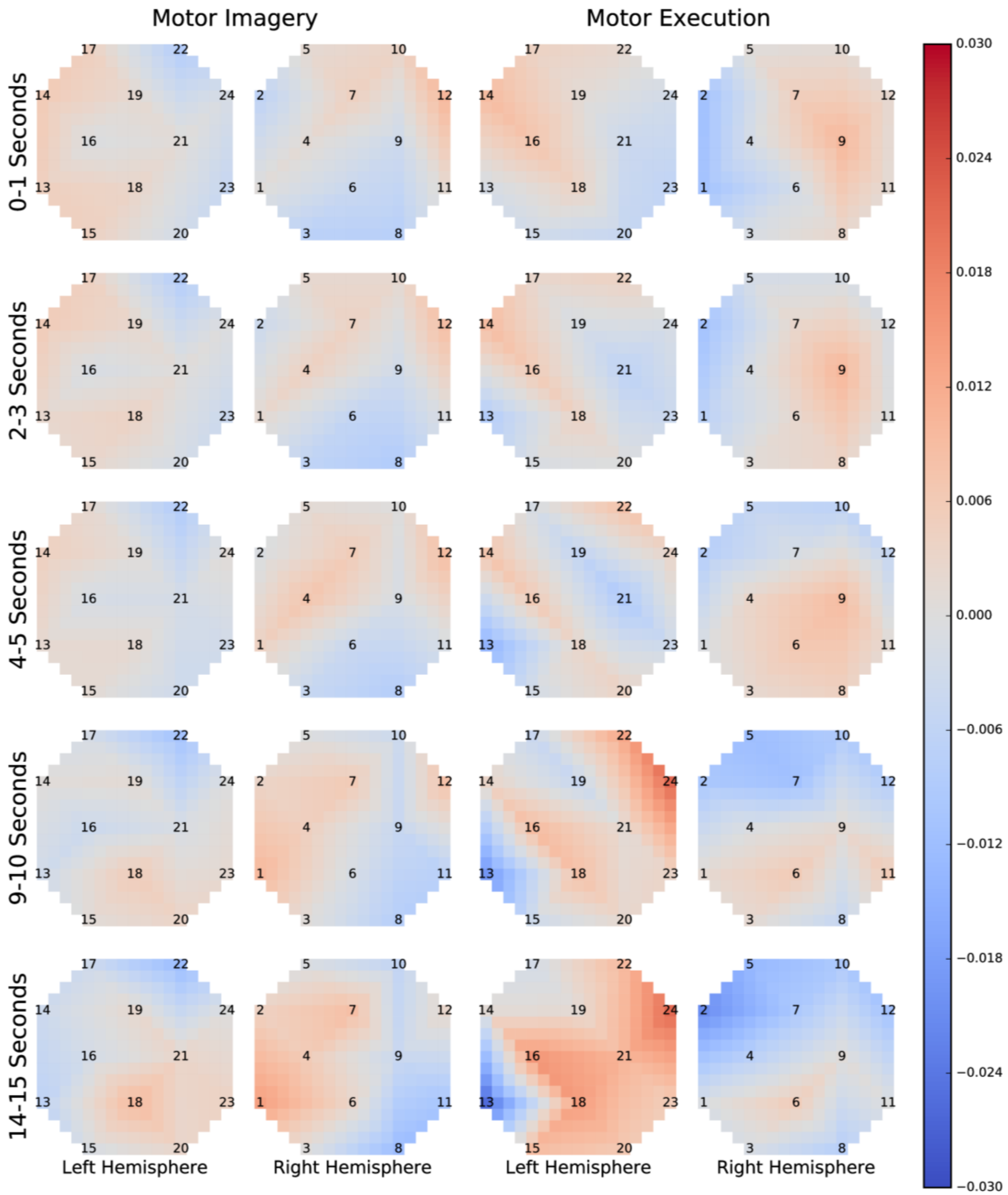


**Fig. S4** Average in HbO activation over time for motor imagery and motor execution during the right foot task
